# Coexistence of Lack of Clinical Manifestation of Oral Mycosis and Systemic Diseases in Edentulous Patients Using Removable Prosthetic Restorations

**DOI:** 10.3390/ijerph17176348

**Published:** 2020-08-31

**Authors:** Izabela Gacon, Aneta Wieczorek

**Affiliations:** Department of Dental Prosthetics, Institute of Dentistry at Jagiellonian University, 31-155 Krakow, Poland; izabela_m@poczta.onet.pl

**Keywords:** candida albicans, stomatitis, systemic diseases

## Abstract

Objective: It is believed that oral infections can increase the risk of systematic diseases, such as atherosclerosis and coronary heart disease, stroke, chronic obstructive pulmonary disease, diabetes, cancer, rheumatoid arthritis, etc. It seems that oral invasive pathogens induce a systemic inflammatory response via mediators released by the cardiovascular system and liver, which increases the risk to the patient of these systematic infections, such as hypertension. On the basis of previous studies of the stomatognathic system, investigating the coexistence of systemic diseases and inflammation in the oral cavity, it can be expected that there is a connection between inflammation of the denture-bearing area in patients using acrylic removable dentures and the presence of systemic diseases, and that patients with inflammation in oral mucosa are more likely to have systemic diseases. Material and method: A retrospective study was carried out on a group of patients seeking prosthetic treatment at the Prosthetic Department of the University Dental Clinic (UKS) from March 2012 to February 2013. All data were collected using a UKS electronic database with KS-SOMED. The minimum period of use for removable prostheses was five years. Results: According to anamnesis, the most common systemic diseases in our study group were hypertension disease. In total, 58% of patients with hypertension disease had no inflammation in the oral cavity. Conclusions: The occurrence of systemic diseases in edentulous people using removable prosthetic restorations, and the subsequent use of medications for these diseases, may result in a lack of clinical symptoms of concomitant fungal infection of the oral mucosa.

## 1. Introduction

Life expectancy in developed countries has increased over the last few decades, a change associated with the significant advances in medicine that offer a wide range of effective therapies for extending human life. Unfortunately, an increase in the length of life does not always involve an increase in its quality. According to the World Health Organization (WHO), tooth decay remains widespread, with an incidence approaching 100% in some countries. Periodontal disease is one of the two most important oral diseases contributing to the global burden of chronic diseases [1]. The incidence of edentulism depends on the region and the country, as well as the age of patients; however, it most often occurs in people over 65 years of age [2]. It is estimated that, by 2025, there will be over one billion people older than 60; by 2050, this group is expected to reach two billion people. The Polish Statistics Office (GUS) predicts that, by 2035, almost one in four Poles will be over 65 years old [3]. Despite increases in life expectancy and continuous improvements in public awareness of oral hygiene, a large percentage of the population will have been supplied with total prostheses. The prolongation of human life is primarily a consequence of the effective treatment of chronic diseases, such as hypertension and diabetes [1]. Aging is a physiological and irreversible process, the progress of which can be modified by many factors throughout life [4], including habits and quality of nutrition, which are in turn affected by the state of the dentition [5,6]. Despite advances in prevention, the dental needs of elderly patients will continue to grow, not only due to the increase in the number of people in this age group, but also due to them preserving more of their natural teeth [7]. Nevertheless, according to Douglass et al. it should not be forgotten that there will still be a large group of older people with edentulism, who will require traditional full dentures [8]. Edentulism and oral diseases are serious problems that are often neglected by patients in both developed and developing countries. The International Classification of Impairments, Disabilities and Handicaps, published by the World Health Organization in 1980 [9], indicates that edentulism can be considered a disability, as it prevents proper biting, chewing and speech. Due to its relationship with the occurrence of general diseases, such as coronary heart disease, stroke and tumors [10], more attention should be paid to the prevention and treatment of edentulism, and to combatting its effects. Anatomical and psychological differences, as well as the coexistence of a number of additional diseases, can make the diagnostics and treatment of edentulism a challenge for both doctors and patients. The study has found that there is also a relationship between inflammation of the oral cavity and certain chronic conditions, such as hypertension [11,12,13]. Systemic diseases, such as oral infections, are associated with assignable risks, including atherosclerosis and coronary heart disease, stroke, chronic obstructive pulmonary disease and diabetes and some more. It seems that oral invasive pathogens induce a systemic inflammatory response via mediators released by the cardiovascular system and liver, which increases the risk to the patient of these infections, such as hypertension [14,15]. Since the inflammation associated with a chronic disorder like periodontopathy has the effect of increasing the risk of some chronic diseases, it is interesting that there is a similar association in the inflammation accompanying denture stomatitis [14,15]. One of the best tools for assessing the clinical assessment of the denture stomatitis is the Newton classification [16]. The need for pharmacological treatment is determined by the growth of colonies of *Candida* spp. fungus.

On the basis of previous studies of the stomatognathic system investigating the coexistence of systemic diseases and inflammation in the oral cavity, it can be expected that there is a connection between inflammation of the denture-bearing area in patients using acrylic removable dentures and the presence of systemic diseases, and that patients with inflammation are more likely to have systemic diseases [14,15].

The aim of this study was to assess the relationship between the incidence of systemic diseases in edentulous individuals using removable prosthetic restorations, and clinical manifestation of concurrent fungal infections of the oral mucosa.

## 2. Material and methods

From March 2012 to February 2013, screening tests were carried out for clinical mucosa examination at the Prosthetic Department of the University Dental Clinic. At this time, all patients who reported for the replacement of prosthetic restorations were being examined. These data were archived to the Kamsoft (Kamsoft, Katowice, Poland) electronic database.

Our retrospective studies used archived data obtained from the above-mentioned examination. In order to minimize patient selection bias, all consecutive patients were entered into the study.

Our study included the data of patients who used removable prosthetic dentures in the upper arch for a minimum of 5 years [16]. The exclusion criteria were smoking, postoperative denture, framework, the lack of a date in the electronic database [16,17,18] and the concomitant presence of two or more diseases. Information on the occurrence of systemic diseases (hypertension, cardiovascular diseases, diabetes, asthma, ulcers, and icterus) was obtained from patients during their medical history [16,18].

The clinical condition of the mucosa in the upper arch was assessed using the Newton classification. In 1962, Newton described [17] the classification for denture stomatitis based exclusively on clinical criteria:

Newton 1: pin-point hyperaemic lesions;

Newton 2: diffuse erythema confined to the mucosa contacting the denture;

Newton 3: inflamatory papillary hyperplasia.

For the purposes of our study, a “zero” group was also created, which included patients with no inflammation of the denture foundation.

All patients who came to the Prosthodontic Clinic in order to receive new prosthetic restorations received an additional obligatory mycological examination before prosthetic treatment began. This examination was performed at 8 a.m. Patients were not to perform any hygienic procedures, nor were they to eat breakfast. Examination was done by a specialist in prosthetic dentistry [16,18]. Immediately after the removal from the mouth of the patient of the upper denture, the doctor swabbed the palate between 2 and 3 palatal folds. The sample was put in a test tube with a Steward transport medium. In the laboratory the sample was incubated for 24 h at 37 °C in Sabouraud medium. The intensity of yeast growth was classified by quantitative assessment as follows [16,19]:

Level 0: lack of fungal growth, up to 10 colonies [16,19];

Level 1: scarce growth, 11–20 colonies;

Level 2: intermediate growth, 21–50 colonies;

Level 3: intense growth, 51–100 colonies;

Level 4: abundant growth, more than 100 colonies.

After mycological examination, antifungal susceptibility was determined for the patients using a fungi test (Bio-Rad, Marnes-la-Coquette, France) and using the disk-diffusion method with Nystatin 100 units (Emapol, Gdańsk, Poland) [16,18].

The results were analyzed as follows:

A. The general health status of patients who did not show symptoms of inflammation of the mucosa (Newton “0”) was compared with that of patients who were clinically symptomatic of inflammation of the denture-bearing area (Newton 1, 2 or 3).
B. The health status of patients who did not show signs of inflammation, but who qualified for treatment, was compared with that of patients with clinically apparent inflammatory symptoms, and who also became eligible for antifungal pharmacotherapy after receiving the results.
C. The health status of the Newton group 0 patients who qualified for pharmacological treatment due to number of fungal colonies was compared with that of the patients in whom symptoms of inflammation of mucosa were visible (regardless of whether they qualified for treatment or not) [16,18].

## 3. Statistical Analysis

Statistical analysis was carried out using R version 3.5.1 (R Core Team (2017). R: A language and environment for statistical computing. R Foundation for Statistical Computing, Vienna, Austria. URL https://www.R-project.org/). The logistic regression was used in the research as a statistical model. A value of *p* < 0.05 was considered significant.

## 4. Results

After applying the inclusion and exclusion criteria, the study group was composed of 279 patients. Of these, 91 (33%) did not show symptoms of the inflammation of the oral mucosa (Newton 0), and 188 patients (67%) had signs of inflammation.

The most common systemic diseases in our study group were firstly hypertension (at 34%), then cardiovascular disease (at 16%). Diabetes was the third most common, being observed in 23 patients (Table 1).

In the first stage, we compared the general health of patients who did not show symptoms of inflammation (“0” in the modified Newton classification) with that of patients who were clinically symptomatic of inflammation in the mucosa (1, 2 or 3 in the Newton classification; see Table 2). During the analysis, a significant statistical difference was found in the occurrence of diseases such as hypertension, diabetes and peptic ulcer disease in the group of patients without clinical manifestations of inflammation in the oral cavity, as compared to those with symptoms of inflammation (Newton 1, 2, 3). In total, 58% of patients with hypertension had no inflammation in the oral cavity.

In the second stage, we compared the health status of patients with no signs of inflammation of oral mucosa, who became eligible for pharmacological treatment upon receiving mycological results, with that of patients with clinically apparent inflammation symptoms, who also became eligible for antifungal pharmacotherapy after receiving the results. In this case (Table 3), a statistically significant difference was found for hypertension. The disease was more common in patients who qualified for pharmacological treatment of the mucosa (over 20 colonies) with a Newton 0 diagnosis than in those with Newton 1, 2 or 3.

In the third stage, the health status of the Newton group 0 patients who qualified for treatment of mucosa was compared with that of the group of patients in whom symptoms of inflammation were visible, regardless of whether they qualified for pharmacotherapy or not. A statistically significant relationship was found for hypertensive and diabetic patients (Table 4). This time, more cases (in %) of hypertension occurred in Newton groups 0 than in 1, 2 and 3.

## 5. Discussion

The increase in length of life does not always involve an increase in its quality. Patients using total dentures are in large part older people suffering from numerous systemic diseases, and thus also from pharmacotherapy. Due to the number of medications used and the diseases that frequently occur, users of acrylic full dentures are exposed to fungal infection. Local factors and systemic factors are among the risk factors for oral mycosis. Systemic factors include hormonal disorders, diabetes, hypothyroidism, hyperparathyroidism, adrenal insufficiency, iron and folic acid deficiencies, immunosuppression, drugs, antibiotics, immune deficiencies and leukocyte dysfunction [14]. Many risk factors can modify an individual’s susceptibility to oral candidiasis, including compromised autoimmunity, tobacco consumption, hyposalivation, denture use, systemic disease, medication [16] and age. Oral infection caused by Candida fungi in healthy people is not a serious disease, because it is a local inflammation and can be treated with locally added medicine, for example Nystatin [19]. However, in cases where patients undergo prolonged antibiotic treatment, and use immunosuppressants or corticosteroids, such an infection may become a systemic fungal infection [19]. Furthermore, in cases of chronic diseases, such as diabetes mellitus, hypothyroidism, hypoparathyroidism, Addison’s disease, Sjögren’s syndrome and others, when saliva secretion is impaired and thus the level of immunoglobulin in saliva decreases, the effectiveness of the humoral immune defense mechanism that controls yeast infection decreases [19]. In addition to reduced saliva flow, poorly-controlled diabetic patients show reduced saliva pH and a reduction of glucose levels, which facilitates oral hypertrophy and colonization [20]. Numerous studies by Czesnikiewicz-Guzik et al. [11,12,13] have found that there is a relationship between inflammation in the oral cavity and certain chronic conditions, such as hypertension. The aim of our study was to assess the relationship between the occurrence of systemic diseases with or without a concomitant infection of the oral mucosa. The results of our study show statistically significant differences in the occurrence of certain diseases, depending on the inflammation present in the clinical trial and on the intensity of the growth, which can serve as an indication to implement antifungal therapy. Hypertension, diabetes and peptic ulcer disease were more frequent in the patients who lacked clinical symptoms of inflammation. It is possible that some of the drugs taken may affect the condition of the mucous membrane, making the symptoms of inflammation invisible in a clinical trial. Patients with a history of peptic ulcer disease did not have their diagnosis documented by gastroscopy, nor was the exact length of the disease activity determined, which means that this group should not be considered in the overall results of the study. Patients with peptic ulcer disease described symptoms such as heartburn, indigestion and abdominal pain after eating meals. The literature describes a relationship between the state of the oral cavity and the occurrence of certain diseases [21]. Czesnikiewicz-Guzik considered the importance of periodontitis as a factor predisposing the individual to cardiovascular diseases. To our knowledge, this study represents the first attempt to assess the relationship between the occurrence of chronic diseases in edentulous people who use removable prosthetic restorations with or without a concomitant infection of the oral mucosa.

Dietrich et al. [22] found that there is evidence to conclude that periodontitis increases the risk of cardiovascular disease, though not in all population groups. Górski and Górska [23] state that, in the light of modern knowledge, periodontitis cannot be considered an independent risk factor for cardiovascular diseases. The study separated patients with hypertension, who were in the primary prevention phase for cardiovascular events, from patients with documented symptomatic cardiovascular disease (myocardial infarction, stroke, or interventions involving the coronary or peripheral arteries). As the results of our work show, patients in the secondary prevention phase for cardiovascular diseases had an asymptomatic state of mycosis that qualified for treatment more often than they had an asymptomatic course of mycosis. This may be associated with the fact that patients with documented cardiovascular disease tend to suffer from advanced systemic inflammation, which impairs the regenerative capacity of the body, and from struggles with opportunistic pathogens. This group was not representative, on account of its small size. The majority of patients with hypertension used statins, with the intention of indefinite use, in order to reduce the high additional risk of cardiovascular events. It should be considered whether statins could mask clinically evident symptoms of opportunistic infections, such as oral mycosis, by reducing inflammation. Statins are widely used to prevent cardiac and cerebrovascular incidents by treating hypercholesterolemia. The anti-inflammatory effect of statins is achieved by acting on many mechanisms of the immune response, among others things, by limiting the number of inflammatory cells in the atherosclerotic plaque and inhibiting the activation of macrophages producing proteolytic enzymes. Besides, statins reduce the production of tumor necrosis factor-alpha (TNF-α) and interferon-gamma (IFNg) in stimulated T cells, and inhibit the immune response of T helper cells (Th-1) [15]. These anti-inflammatory effects may be potential statin mechanisms for reducing oral mucosal infection. Studies have shown the role of statins in maintaining microvascular integrity and restoring normal endothelial function, as well as in inhibiting cell adhesion molecules [24]. Statins can, therefore, play an essential role in the early course of mucositis. Besides, due to the inhibition of HMG-CoA reductase, which affects the synthesis of ergosterol, statins have shown a direct antifungal effect. Statins can also cause deletions in the yeast mitochondrial genome, impeding their development [25]. Additionally, even at clinically unachievable levels, synergy was observed between statins and fluconazole [24]. Another single-center study [26] did not show any benefits in the results from the treatment of candidates with statins. It was a study in which only 14 cases of statin users were analyzed. Statins have been extensively studied in the context of bone regeneration in periodontal disease, but soft tissue healing remains relatively less studied [27]. Further statin studies should be considered as a potential adjunct therapeutic strategy with a positive effect on the healing of hard and soft periodontal tissues. Additionally, the effect of statins on proresolution molecules has not been studied in the context of wound healing or periodontal regeneration. Since not all available statins have been tested, future research must assess the effects of these drugs on antibacterial, anti-inflammatory, immune and osteoprogenitor responses. In summary, choosing the optimal dose of statins, based on the drug delivery method and carrier used, can increase the positive effect of statins on periodontal treatment results. Besides, combining statins with growth factors or other drugs in an efficient carrier system may be beneficial in promoting periodontal regeneration [27]. Further research is needed focusing on the possible effect of statins in masking symptoms of fungal infection. We did not find in the literature any comparisons of the incidence of systemic diseases depending on the visible or invisible clinical symptoms of fungal infection.

The strength of this study is the number of study participants. Its limitations are the lack of participants without comorbidity, and the lack of a uniform distribution of participants with systemic diseases.

## 6. Conclusions

The occurrence of systemic diseases in edentulous people using removable prosthetic restorations and the subsequent use of medications for these diseases may result in the lack of clinical symptoms of concomitant fungal infection of the oral mucosa.

## Figures and Tables

**Table 1:** The general health of patients.

Diseases	Percent (Number) of Patients
Hypertension	34% (96)
Cardiovascular diseases	16% (46)
Diabetes	8% (23)
Asthma	2% (5)
Ulcers	3% (8)
Icterus	1% (3)
Total	100% (279)

**Table 2:** The general health of patients without inflammation (Newton 0) and with inflammation of oral mucosa—Newton 1, 2, 3.

Disease	Newton 0	Newton 1,2,3	OR	*p*
Hypertension	53 (58%)	43 (23%)	0.213	<0.001 *
Cardiovascular diseases	20 (22%)	26 (14%)	0.57	0.088
Diabetes	13 (14%)	10 (5%)	0.337	0.014 *
Asthma	3 (3%)	2 (1%)	0.315	0.211
Ulcers	7 (8%)	1 (1%)	0.064	0.011 *
Icterus	3 (3%)	0 (0%)	0	0.996

* statistically significiant.

**Table 3:** The health of the patients who became eligible for antifungal pharmacotherapy in group Newton 0 and Newton 1, 2 or 3.

Disease	Newton 0	Newton 1,2,3	OR	*p*
Hypertension	20 (22%)	32 (17%)	0.728	0.32
Cardiovascular diseases	4 (4%)	18 (10%)	2.303	0.142
Diabetes	7 (8%)	7 (4%)	0.464	0.163
Asthma	2 (2%)	2 (1%)	0.478	0.465
Ulcers	1 (1%)	1 (1%)	0.481	0.607

**Table 4:** The health status of the Newton group 0 patients who qualified for treatment compared with the group of patients in whom symptoms of inflammation were visible—Newton 1, 2 and 3.

Disease	Newton 0	Newton 1,2,3	OR	*p*
Hypertension	20 (22%)	43 (23%)	1.053	0.867
Cardiovascular diseases	4 (4%)	26 (14%)	3.491	0.024 *
Diabetes	7 (8%)	10 (5%)	0.674	0.44
Asthma	2 (2%)	2 (1%)	0.478	0.465
Ulcers	1 (1%)	1 (1%)	0.481	0.607

* statistically significiant.
